# Brain age and other bodily ‘ages’: implications for neuropsychiatry

**DOI:** 10.1038/s41380-018-0098-1

**Published:** 2018-06-11

**Authors:** James H. Cole, Riccardo E. Marioni, Sarah E. Harris, Ian J. Deary

**Affiliations:** 1 Department of Neuroimaging, Institute of Psychiatry, Psychology & Neuroscience, King’s College London, London, UK; 2 Computational, Cognitive and Clinical Neuroimaging Laboratory, Department of Medicine, Imperial College London, London, UK; 3 Centre for Cognitive Ageing and Cognitive Epidemiology, University of Edinburgh, Edinburgh, UK; 4 Centre for Genomic and Experimental Medicine, MRC Institute of Genetics & Molecular Medicine, University of Edinburgh, Edinburgh, UK; 5 Department of Psychology, University of Edinburgh, Edinburgh, UK

**Keywords:** Neuroscience, Predictive markers, Genetics, Biological techniques

## Abstract

As our brains age, we tend to experience cognitive decline and are at greater risk of neurodegenerative disease and dementia. Symptoms of chronic neuropsychiatric diseases are also exacerbated during ageing. However, the ageing process does not affect people uniformly; nor, in fact, does the ageing process appear to be uniform even within an individual. Here, we outline recent neuroimaging research into brain ageing and the use of other bodily ageing biomarkers, including telomere length, the epigenetic clock, and grip strength. Some of these techniques, using statistical approaches, have the ability to predict chronological age in healthy people. Moreover, they are now being applied to neurological and psychiatric disease groups to provide insights into how these diseases interact with the ageing process and to deliver individualised predictions about future brain and body health. We discuss the importance of integrating different types of biological measurements, from both the brain and the rest of the body, to build more comprehensive models of the biological ageing process. Finally, we propose seven steps for the field of brain-ageing research to take in coming years. This will help us reach the long-term goal of developing clinically applicable statistical models of biological processes to measure, track and predict brain and body health in ageing and disease.

## Introduction

As we age, the molecules, cells, tissues, and organs within our bodies undergo changes. The biology of the ageing process is complex [1], and is yet to be fully characterised. Though precise definitions of ageing can be controversial, it can generally be seen as the gradual accumulation of deleterious biological changes accompanying a progressive loss of function [2]. What is uncontentious, however, is that ageing increases the risk of morbidity and mortality in humans, as in most species (c.f., the polyp species *Hydra*). What is also clear is that humans do not experience biological ageing at the same rate, with pronounced differences in the outward manifestations of ageing being observed (e.g., hair loss, skin wrinkles, presbyopia). The age of onset for age-related diseases is also highly variable, as is individual lifespan. This has motivated biogerontological research efforts to measure ageing from a biological perspective, with the goal of producing ‘ageing biomarkers’ (see Box 1) that are better predictors of disease risk and residual lifespan than chronological age alone [3]. In theory, such a biomarker could be used to predict risk of age-related disease and mortality, to monitor biological ageing over time, and to evaluate potential treatments aimed at improving health during ageing.

Ageing has consequences for the brain and any patient suffering from a chronic psychiatric or neurological disorder will be exposed to ageing effects during the course of their disease. Potentially, by measuring the biological age of the brain in people with neuropsychiatric diseases, we may better understand disease risk and resilience, the effects of these diseases on the ageing brain, and improve predictions of health outcomes by capturing individual differences in the interactions between ageing and disease. The physiological changes associated with brain ageing include the macroscopic (e.g., ventricular enlargement, cortical thinning, decreased post-mortem weight, the accumulation of white matter hyperintensities) [4–6], the cellular (e.g., synaptic pruning, axonal loss, mitochondrial changes, alterations to glial cell numbers) [7–9], through to the molecular (e.g., altered gene expression, disrupted calcium signalling, epigenetic changes) [9–11]. Behaviourally, brain ageing is associated with cognitive decline (commonly described as cognitive ageing; particularly affecting cognitive domains such as information processing speed, memory, reasoning, and executive function) [12, 13], decreased well-being and increased symptoms of low mood [14–16]. Neurodegenerative diseases are also increasingly common in older adults [17], with dementia arguably representing the final common endpoint of various age-related neuropathological insults.

The importance of maintaining a healthy brain during ageing is increasingly being recognised as a goal for society. Not only is the risk of neuropsychiatric diseases increased, but symptoms are exacerbated and prognosis worsened by older age. Accordingly, neuroscientists have taken a biogerontological approach to develop specific in vivo biomarkers to measure brain ageing [18–22]. Here, we outline how these putative brain-ageing biomarkers could potentially provide information about how the brain changes with age and how neuropsychiatric diseases interact with the brain-ageing process. Building on our recent opinion article [23], here we aim to put brain-ageing biomarker research in the context of more classic biogerontological research into blood-derived or physiological ageing biomarkers. Finally, we discuss how and why brain and other bodily ageing biomarkers should be combined, as this may provide insights into more fundamental aspects of biological ageing as well as help predict neuropsychiatric and general health outcomes.

Box 1 Ageing biomarkers The rationale for research into ageing biomarkers is as follows: (i) pronounced individual differences in lifespan are commonly seen in humans, suggesting that chronological time is not directly equivalent to the rate of ageing; (ii) differences in ageing rates are probably mediated by underlying biological processes that influence lifespan and age-related disease manifestation, themselves influenced by genetic factors and environmental conditions; (iii) as human lifespan exceeds the reasonable timescale of interventional trials for improving healthy ageing, methods for accurately measures ageing rates are needed (i.e., ageing biomarkers).

As pointed out by Ludwig & Smoke in 1980 [28], biological age is not something that can be directly observed; instead it must be inferred from quantifiable epiphenomena. These quantifiable epiphenomena (i.e., ageing biomarkers), have long been sought by researchers in the field of biogerontology and take many forms. Candidates include grip strength, gum health, lung function, HbA1C levels, mean arterial pressure, white blood cell count, cell membrane viscosity, corneal endothelial thickness, cholesterol levels or cytomegalovirus optical density, to name but a few. As detailed herein, recent ‘omics research has identified further candidates using profiles of epigenetic, metabolomic, or transcriptomic signatures [69]. More recently, neuroscientific measures derived from neuroimaging are also being considered [23, 128].

Given the plethora of candidates, efforts to standardise the criteria for qualification as an ageing biomarker have been proposed. For example, as reported by Johnson [36], the American Federation for Aging Research stipulated that an ageing biomarker must:

1. Predict the rate of ageing, (i.e., inform where a person is in their total lifespan). It must be a better predictor of lifespan than chronological age.
2. Monitor a basic process that underlies the ageing process, not the effects of disease.
3. Can be tested repeatedly without causing harm.
4. Work in humans and in laboratory animals, so it can be tested in animals prior to validation in humans.

However, as noted by both Johnson and Sprott [36, 129], the second criterion is somewhat contentious. Given that ageing is the major risk factor for many diseases, disentangling which processes are distinct to ageing or to disease, and particularly pre-manifest disease, is extremely challenging. In fact, some ageing and disease processes may well be shared, only differing in degree rather than in form.

Nevertheless, the search for ageing biomarkers has continued apace. Although some studies frame a measure of a single aspect of human biology as a marker of biological age (i.e., implying that this is a global measure for an individual), it appears increasingly unlikely that any such universal biological age measure will be identified [121]. Instead, the growing consensus is on combining data from different aspects of biology to generate composite measures that better reflect a unitary process [37, 119, 120]. In future, it is hoped that increasingly well-characterised datasets containing multiple candidate ageing biomarkers will allow the modelling of both local biological age (e.g., brain age, leukocyte telomere age, blood-cell DNA methylation age) and global, composite biological age. This should enable us to reach the optimal panel of markers of underlying patterns of biological ageing, that can be used for trialling interventional strategies and predicting future age-related health.

## Brain ageing, cognitive ageing, and physiological ageing

**Fig. 1:**
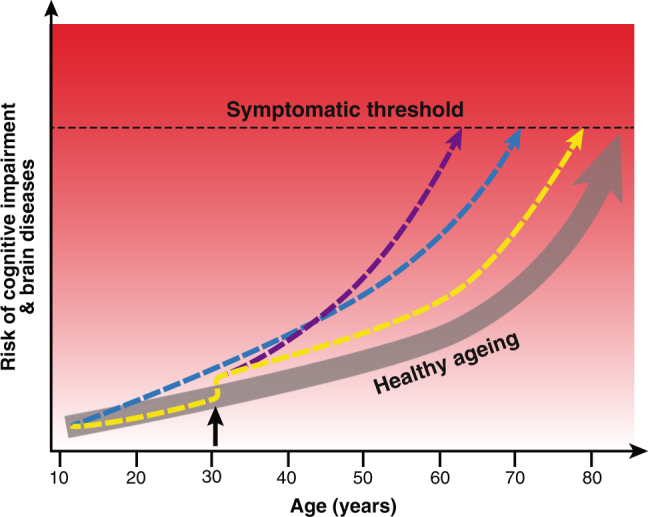
Differential trajectories of brain ageing. Illustration of the concept of ageing trajectories, specifically brain ageing. With increasing age, even healthy people are at higher risk of cognitive impairment and brain diseases, eventually reaching a threshold where symptoms appear. Individuals can differ in their brain-ageing trajectories. For example, a person may have genetic or developmental environmental factors that confer a higher rate of ageing throughout life (blue line). Alternatively, someone may experience a traumatic injury or infection in adulthood (black arrow), which results in them following an accelerated (purple line) or accentuated, but stable (yellow line), trajectory of brain ageing. While the example used here is of brain ageing, the same model can be used to conceptualise biological ageing more generally

Neuroimaging, and particularly magnetic resonance imaging (MRI), can provide varied and detailed information on the living human brain. The application of methods derived from the study of artificial intelligence, particularly machine learning, has enabled researchers to use high-dimensional MRI datasets to build predictive statistical models of brain ageing. These models assume a trajectory of brain ageing (Fig. 1) that represents an individual’s accumulation of deleterious changes that lead to alterations in brain function and increased risk of cognitive decline and disease.

**Fig. 2:**
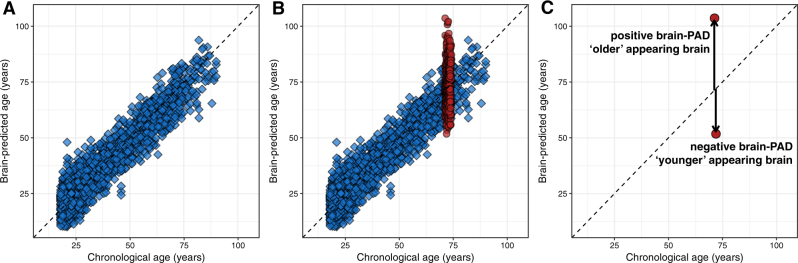
Brain-predicted age and brain-PAD. **a** The results of using Gaussian processes regression to predict chronological age using structural neuroimaging data in a sample of 2001 healthy individuals, aged 18–90 years, based on ten-fold cross validation (mean absolute error = 4.93 years, *r* = 0.94). **b** Same as **a**, with the brain-predicted age values for participants from the Lothian Birth Cohort 1936 overlaid in red. This demonstrates that, despite the narrow age range at time of scanning (72–74 years), these *N* = 669 individuals had a wide variability in brain-predicted ages. **c** Illustration of how brain-predicted age difference (brain-PAD) scores are calculated, highlighting the individuals from the Lothian Birth Cohort 1936 with the lowest and highest brain-predicted ages. Brain-PAD is the difference between brain-predicted age and chronological age for an individual. Positive brain-PAD suggests that the individual’s brain appears older than their chronological age, whereas a negative brain-PAD suggests that their brain appears younger

The models, generally using data from T1-weighted MRI scans of brain structure, are informed by ‘learning’ the relationship between age and brain structure in large samples of healthy adults. Model performance, evaluated by predicting chronological age based on brain scans from new individuals, results in mean prediction errors of less than five years [19, 21, 24–27]. These models can then be used to generate a biological age from neuroimaging data, a ‘brain-predicted age’. Following the established biogerontological model of determining the discrepancy between the chronological and biological age of an organism [28], if an individual’s brain-predicted age is greater than their chronological age, this indicates that their brain structure more closely resembles a healthy person who is older than they are. The assumption is that greater discrepancies between brain-predicted age and chronological age reflect poorer brain health, for a given age. Different research groups refer to this discrepancy using different names (e.g., brain-age gap [G_BA_], brain-age gap estimate [brainAGE]) [21, 29]; here, we use the term brain-predicted age difference (brain-PAD); mathematically this is chronological age subtracted from brain-predicted age (Fig. 2).

This approach to modelling brain ageing makes inferences based on errors in model prediction, hence external validation is essential. Key to validating brain-predicted age is to relate it to other measures of ageing, such as cognitive or physiological assessments. Using data from the Lothian Birth Cohort 1936 [30, 31], we recently sought to do this. Using neuroimaging measures of brain volume from this general population, narrow age-range (approximately 73 years old) cohort, brain-PAD was calculated for 669 people [32]. We demonstrated that brain-PAD was significantly negatively related to a composite measure of cognitive function (standardised beta from linear regression = −0.12), designed to reflect ageing-related changes in general, fluid cognitive functioning [33]. Furthermore, in this cohort, brain-PAD was also related to a panel of physiological measures designed to assess health in ageing. Having an older-appearing brain was associated with weaker grip strength (standardised beta = −0.06), poorer lung function (standardised beta = −0.07), and slower time to walk 6 metres (standardised beta = 0.13). This suggests that measures of age-associated brain volume are sensitive to the same underlying factors that cause physiological changes during ageing.

Another potential demonstration of the validity of an ageing biomarker is to consider how such a measure behaves in circumstances of ‘accelerated’ biological ageing. One such example is Down’s syndrome (DS), where trisomy of chromosome 21 results in a broadly progeroid (i.e., resembling older age) phenotype [34, 35]. Our analysis of brain-predicted age in 46 adults with DS showed that the mean brain-PAD was 2.5 years in this group, significantly greater than local healthy control participants (mean brain-PAD = −5.2 years). This suggests that some of the changes to brain structure in DS resemble those seen during ageing, much like the outward physiological manifestations of the syndrome. When considering the variability of brain-PAD scores in participants with DS, those with greater brain-PAD had greater levels of beta-amyloid deposition (measured using Pittsburgh-compound B [PiB] positron emission tomography [PET] scans, standardised beta = 0.29). Interestingly, in individuals with DS who also had signs of amyloid deposition, there was a strong relationship between brain-PAD and cognitive performance (standardised beta = −0.51). This was not seen in DS individuals who were ‘PiB negative’ for amyloid. In other words, people with DS who exhibited signs of some of the key pathological facets of brain ageing (i.e., amyloid deposition, cognitive decline) also had older-appearing brains. This suggests that brain-PAD is a potentially useful method for understanding individual differences in brain ageing in DS, which may in turn relate to risk of Alzheimer’s disease. However, the large effect sizes observed in this relatively small study require replication and longitudinal assessment before brain-PAD can be taken further towards clinical applications in DS.

**Fig. 3:**
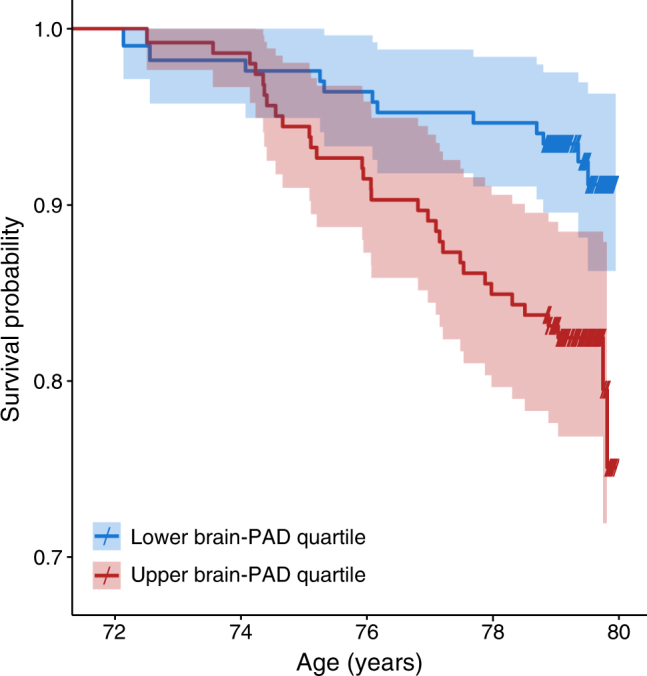
Survival curves based on high and low brain-PAD at age approximately 73 years. Illustration of the relationship between brain-predicted age difference (brain-PAD) and survival over 8 years after MRI scanning at a mean age of 73 years in the Lothian Birth Cohort 1936 [32]. Kaplan–Meier plot shows the survival curves for individuals grouped according to whether their brain-PAD was in the upper (red) or lower (blue) quartile of the distribution. Survival probably is observed to be lower for those with high brain-PAD. Crosses on the survival curves indicate age at last assessment (i.e., right censored data). These are for illustration only; the analyses were conducted with all-available participants’ data, and brain-PAD was entered as a continuous variable

The relationship of a putative ageing biomarker to residual lifespan (i.e., how much longer a person has to live) is another important measure of validity [36]. Again, using the Lothian Birth Cohort 1936, we assessed whether brain-PAD related to survival after MRI scanning at age ~73 years. Mortality data up to age ~80 years were obtained via linkage with healthcare records in Scotland. At time of analysis, 10.9% (*n* = 73 out of 669) of the participants had died. Here, brain-PAD was a significant predictor of survival in a Cox proportional hazards regression model, whereby having an older-appearing brain was associated with reduced survival times (Fig. 3). Specifically, for each additional year of brain ageing (+1 year brain-PAD) there was a 6% increase in the likelihood of death (hazard ratio = 1.06, 95% confidence intervals 1.03–1.09) [32]. Interestingly, the most common cause of death was cardiac disease, suggesting that brain ageing is not causing death per se, but that the brain is sensitive to the deleterious consequences of ageing that occur more systemically. As Jackson et al. [37] pointed out, “while the selected biomarkers are not thought to be specific harbingers of death, their rates of decline are assumed to reflect a decline in organ and system efficiency, and hence to qualify for the purpose in hand”. Potentially, neuroimaging-derived brain-predicted age may be a proxy of underlying brain and systemic integrity during ageing.

Though initial data validating brain-predicted age as an ageing biomarker are promising, additional validation in other age-related contexts are needed to support its wider use in brain diseases. Brain-predicted age is under a degree of genetic influence [19], which motivates the search for relevant risk genes for poorer brain ageing. Brain-predicted age is reliable (intra-class correlation coefficient = 0.97 within scanner, 0.92 between scanner) [19], making it suitable for use in longitudinal studies predicting individual trajectories of brain health over time. Given suitable development, brain-predicted age may in future provide insights into overall biological ageing across the population (see Box 2).

Box 2 Seven future directions for brain-ageing research The overall goals of brain-ageing research are: (i) to understand how the brain changes as we age; (ii) to understand why the brain changes as we age; and (iii) to help improve brain health and reduce the impact of cognitive decline and neuropsychiatric diseases during ageing. To meet these goals, we have identified seven key future directions for the field:

1. *Characterise the spectrum of brain ageing*. To better understand how the human brain changes with age, we need a clearer picture of the different possibilities. Efforts to define the population variability in brain structure and function across the life course and to build so-called normative models of brain ageing will be vital to define this spectrum of different possibilities. This will enable us to locate an individual within the spectrum of brain ageing and have a clearer idea of what constitutes the gradient between healthy and pathological ageing-related changes that may result from a neuropsychiatric disorder.
2. *Continue to open up more, expertly curated, datasets*. The number of large datasets becoming publicly-available is increasing, and the informatics necessary for processing and analysing these data to maximise their utility are also emerging. As even more biomedical data sharing occurs, it is essential that we further encourage comprehensive, scalable and extendable informatics platforms and standards. Efforts such as the Dementias Platform UK (https://www.dementiasplatform.uk/) and BRAINS Imagebank (http://www.brainsimagebank.ac.uk/) are underway to collate data from diverse sources [130]. Standardisation initiatives such as BIDS (Brain Imaging Data Structure: http://bids.neuroimaging.io) and analytic platforms such as Nipype (http://nipype.readthedocs.io) are gathering more traction in the community. Such efforts should allow sharing and combining of different datasets from diverse sources and will be particularly important as moves to open access to clinically acquired neuroimaging data gather momentum.
3. *Externally validate brain-ageing models*. Eminent statistician George Box said, “Essentially, all models are wrong, but some are useful”. For brain-ageing research, along with any other statistical modelling endeavour, it is essential that predictive models are validated in external datasets to demonstrate that they provide useful predictions. Merely showing reasonable within-sample accuracy is insufficient, particularly if framed around unilluminating prediction goals (e.g., classification of patients with manifest disease from healthy controls).
4. *Identify individual spatial patterns of brain ageing*. Currently, most brain-age research has taken a global approach, using either whole brain or total grey matter data to generate a single brain-predicted age value per individual. However, it is likely that differential spatial patterns of brain ageing occur within individuals, e.g., in the context of different diseases. Therefore, research aimed at developing individualised ‘saliency’ maps to better understand which parts of the brain are driving brain-age predictions or even voxelwise brain-predicted age images could be informative. Such research could be used to compare spatial patterns of brain ageing across different disease states, potentially distinguishing them or identifying within-disease subgroups, leading to new insights about the brain regions underlying healthy ageing or ageing-disease interactions.
5. *Recognise the continuum of ageing and disease*. Conventional wisdom states that health and disease are qualitatively different, and that ageing itself is not a disease. However, in the modern era of chronic disease and extended pre-manifest disease stages, this distinction needs to be reconsidered. Instead of measuring how healthy people and people with a disease differ, we need a conceptual shift, so we can better consider how ageing and disease processes are similar, and how they might interact to influence prognosis and treatment response in the case of neuropsychiatric and neurodegenerative diseases.
6. *Integrate more closely with biogerontology*. As we outline herein, evidence now suggests that, to gain a comprehensive understanding of ageing from a biological perspective, data from all-available sources needs be considered. Historically, the neuroscientific domain of brain ageing has been separated from that of biogerontology, where the brain is largely neglected. Given the importance of brain–body interactions for mediating a host of biological functions and behaviours, it essential that brain ageing and biogerontological research become more closely integrated, through collaboration and research capacity building, to the benefit of both.
7. *Aim for greater clinical applicability.* A greater focus on clinical and healthcare applications is needed to translate this growing knowledge of brain ageing to have practical utility. Given the ageing global population, the prevalence of chronic diseases that influence brain health, and the costs to society associated with cognitive decline and dementia, we urgently need to improve brain and body health as we age. Predictive models of brain and body ageing could be beneficial here. As indices of underlying biological ageing, they could serve as either a measurable risk factor for increased susceptibility to age-related health problems or a surrogate outcome measure of protective or deleterious effects of disease or interventions on the ageing brain. For example, in the context of clinical trial of a neuroprotective treatment in a neuropsychiatric disease, these measures could be used to stratifying enrolment, screening for people with higher biological age, under the assumption that they would be at greater risk for clinical decline over a shorter timeframe. Longitudinal changes to measures of biological age could also be used as outcomes in clinical trials, particularly in at-risk or prodromal groups where measuring clinical symptoms is challenging. Evidence that an intervention reduces biological age differences over time could suggest that a given intervention is beneficial to brain health. Finally, biological measures of brain and body have the potential be used in general clinical practice, to screen people and identify those at increased risk of poorer brain and body health as they age.

## Brain ageing and disease

Ageing is a key risk factor for many major medical health problems, not least neurodegenerative diseases. Furthermore, even if ageing does not increase risk of a specific chronic disease, older age likely worsens disease symptoms and prognosis. This motivates research into how measures of biological ageing relate to disease risk or disease progression, and how diseases in turn can influence rates of biological ageing. In fact, a number of neurological and psychiatric diseases have been proposed to result in premature or accelerated ageing, based on clinical observations and behavioural or biological research. These include schizophrenia, depression, epilepsy, HIV infection, and traumatic brain injury [38–42]. Validating these ideas is currently difficult, as there is no consensus operationalised definition of an accelerated-ageing phenotype, making the concept controversial [43]. Nevertheless, neurodegeneration is one of the four key components of ageing proposed by Margolick & Ferruci [43]; therefore, models of brain ageing offer a possible window into the relationship between ageing and disease. If a disease can be shown to accelerate the ageing-related phenotype of brain structure, this provides information about the potential mechanisms involved and highlights possible commonalities across diagnostic categories. Importantly, it also enables the measurement of individual differences in disease groups, with prognostic implications for future brain health.

A number of psychiatric disorders have been investigated using brain-predicted age metrics (Table 1). In schizophrenia, reports have suggested that not only is greater brain ageing observed, particularly in males [24, 44], but that this accelerates over time [29]. In other psychotic conditions, such as bipolar disorder and at-risk mental states, increased brain ageing is less evident [24, 44]. Koutsourleris et al.'s comprehensive study also included patients diagnosed with major depressive disorder (MDD), finding a mean added brain ageing of 4 years [24]. Individuals with borderline personality disorder were studied, showing a mean added brain ageing of 3.1 years. These preliminary studies indicate that psychiatric disorders do indeed result in premature age-related changes to brain structure, though further validation in larger samples is required, such as that currently being undertaken by the ENIGMA-MDD consortium [45].

**Table 1:** Studies of brain-predicted age in disease

Study	Clinical group	*N*	Age (mean ± SD, or range)	MRI features	Algorithm	Brain-PAD (mean, years)
Psychiatric disorders						
Franke et al. [21]	Alzheimer’s disease	102	76 ± 8	GM	RVR	10.0
Franke and Gaser [46]	MCI—stable	36	77 ± 6	GM	RVR	BL: −0.5 FU (3 yrs): −0.4
	MCI—progressive	112	74 ± 7	GM	RVR	BL: 6.2 FU (3 yrs): 9.0
	Alzheimer’s disease	150	75 ± 8	GM		BL: 6.7 FU (2 yrs): 9.0
Koutsouleris et al. [24]	High psychosis risk	89	25 ± 6	GM	SVR	1.7
	Schizophrenia	141	28 ± 12	GM	SVR	5.5
	Major depression	104	42 ± 8	GM	SVR	4.0
Gaser et al. [49]	MCI—progressive (early/late)	58/75	74 ± 7/75 ± 7	GM	RVR	8.7/5.6
Schnack et al. [29]	Schizophrenia	341	34 ± 12	GM	SVR	BL: 3.4 FU (4 yrs): 4.3
Löwe et al. [48]	MCI—stable (APOE ε4 carriers/non-carriers)	14/22	77 ± 6/77 ± 6	GM	RVR	BL: −0.9/−0.9 FU(3 yrs): 0.0/−0.6
	MCI—progressive (APOE ε4 carriers/non-carriers)	78/34	74 ± 6/75 ± 9	GM	RVR	BL: 5.8/5.5 FU (3 yrs): 8.7/7.3
	Alzheimer’s disease (APOE ε4 carriers/non-carriers)	101/49	74 ± 7/76 ± 9	GM	RVR	BL: 5.8/6.2 FU (2 yrs): 8.3/7.7
Nenadic et al. [44]	Bipolar disorder	22	38 ± 11	GM	SVR	−1.3
	Borderline personality disorder	57	26 ± 7	GM	SVR	3.1
	Schizophrenia	45	34 ± 10	GM	SVR	2.6
Li et al. [47]	Alzheimer’s disease	411	75 ± 7	Hippocampal volume	SVR	7.0
Varikuti et al. [66]	Alzheimer’s disease	163	56–91	GM	LASSO	8.5; 10.7^a^
	MCI	64	55–87	GM	LASSO	6.2; 5.4^a^
Kolenic et al. [131]	Psychosis (first episode)	120	27 ± 4.9	GM	RVR	2.6
Guggenmos et al. [132]	Alcohol dependence	119	20–65	GM	MLRR	4.0
Neurological disorders						
Cole et al. [52]	Traumatic brain injury	99	38 ± 12	GM/WM	GPR	4.7/6.0
Cole et al. [59]	HIV	162	57 ± 8	Whole brain	GPR	2.2
Cole et al. [133]	HIV	131	56 ± 6	Whole brain	GPR	BL: 1.6 FU (2 yrs): 1.6
Cole et al. [50]	Down’s syndrome	46	42 ± 9	Whole brain	GPR	2.5
Pardoe et al. [58]	Epilepsy (medically refractory/newly-diagnosed)	94/42	32 ± 14/31 ± 11	Whole brain	GPR	4.5/0.9
Liem et al. [53]	Objective cognitive impairment (mild/major)	632/251	58 ± 15/58 ± 16	Whole brain	SVR/RF	0.7/1.7
Physiological disorders						
Franke et al. [60]	Diabetes (type II)	98	65 ± 8	GM	RVR	4.6
	Diabetes (type II)—longitudinal	12	63 ± 7	GM	RVR	BL: 5.1 FU (4 yrs): 5.9
Ronan et al. [61]	Obesity	227	58 ± 17	WM	NLME	10.0
Franke et al. [134]	Gestational nutrient restriction (female/male)	22/19	67 ± 0.2/67 ± 0.1	GM	RVR	0.9/2.5

*BL* baseline, *FU* follow-up, *GM* grey matter, *GPR* Gaussian process regression, *LASSO* Least Absolute Shrinkage and Selection Operator, *MCI* mild cognitive impairment, *MLRR* multi-linear ridge regression, *NLME* non-linear mixed effects model, *RF* random forests, *RVR* relevance vector regression, *SVR* support vector regression, *WM* white matter

^a^Study included results from two different training datasets

The development of mild cognitive impairment (MCI) and subsequent Alzheimer’s disease are some of the key pathological consequences of the brain-ageing process. Using neuroimaging, people diagnosed with Alzheimer’s have been shown to have greater apparent brain ageing, from various analyses using the Alzheimer’s Disease Neuroimaging Initiative (ADNI) dataset [21, 46–48]. In people with MCI, brain-predicted age was a significant predictor of progression to dementia within three years of baseline MRI scan [46, 48, 49]. This is important as it demonstrates that brain-predicted age is sensitive to subtle underlying changes to the brain that occur prior to overt disease manifestation. In slowly progressive neurodegenerative conditions like Alzheimer’s, tools that identify people at greater risk of future morbidity could be particularly useful, both for clinical practice and for the design of clinical trials, either by stratifying trial enrolment or as a surrogate outcome measure. Some evidence also suggests that *APOE* genotype is associated with increased brain ageing in ADNI [48], with Alzheimer’s disease patients carrying the e4 allele showing greater longitudinal changes in brain-predicted age compared to non-e4 carriers. However, an *APOE* effect was not observed in people with DS nor in a general population sample [32, 50]. Whereas neuroimaging measures of brain ageing appear to be heritable [19], as would be expected given the demonstrated heritability of measures of brain volume [51], further research is necessary to conclusively identify specific genetic factors that influence rates of brain ageing.

An important consideration regarding the relevance of brain-predicted age for psychiatric diseases is the lack of specificity. The average ‘added’ brain-ageing is relatively similar across different disorders. The limits the use of brain-predicted age to differentially diagnose diseases or generate disease-specific insights into potential brain-structural mechanisms. An alternative application of brain-predicted age (or other ageing biomarkers) in the context of psychiatric diseases is to capture individual differences within disease groups, as the observed variability is often relatively high. For example, if within a group of patients with MDD some individuals show higher brain-PAD than others, these patients may have experienced greater severity of disease, resulting in greater downstream accumulation of age-related damage to the brain. They may also be at increased risk of subsequent general cognitive dysfunction, particularly given reports associating brain-predicted age with cognitive performance [32, 50, 52, 53]. As cognitive dysfunction is thought to relate to general functional ability in MDD [54] and a combination of MDD and cognitive decline may increase risk for dementia in older adults [55–57], then information about an individuals’ apparent brain ageing could be used to target treatments and interventions to those at greater risk.

Neurological conditions have also been assessed to establish their influence on brain ageing (Table 1). Increased brain ageing has been observed in survivors of a traumatic brain injury [52], in treatment-resistant epilepsy [58], and to a lesser extent, in adults with successfully treated HIV [59]. As with studies of people from the general population, these studies showed a moderate, but consistent relationship between brain-predicted age and cognitive performance, whereby individuals with older-appearing brains performed more poorly in a range of cognitive domains. So, although accentuated in certain disease conditions, it seems that measures of brain ageing are tapping into something more general that relates to brain health. Future research should follow these groups longitudinally to establish whether the apparent increases in brain ageing are stable or accelerating over time, and how this relates to cognitive outcomes.

Finally, the relationship between physiological health conditions without an explicit central nervous system (CNS) component and brain ageing has been explored. Both obesity and type II diabetes mellitus have been associated with higher brain-predicted age than chronological age [60, 61]. These studies reinforce two important points. Firstly, they support the idea that chronic systemic disorders have wide-ranging deleterious effects on the body, which includes affecting the brain, leading to increased accumulation of age-like changes. Secondly, technical developments in neuroimaging mean that we now have a robust and reliable measure of the adverse effects of these conditions during ageing. For example, in light of concerns over the chronic neurological consequences of obesity for an ageing society [62], brain-predicted age could be used to measure individual differences in how severely obesity impacts brain health. Of course, it will be useful to see future studies like these including other ageing biomarkers so that brain ageing can be compared with them in different contexts.

Whether or not ageing itself is a disease is a controversial topic [63]. Nevertheless, the growing body of literature investigating neuropsychiatric diseases from the perspective of brain ageing suggests that these diseases can accentuate some of the brain structural changes that occur in healthy ageing. However, it is important not to assume that the same effects are occurring in different conditions in which brain-predicted age is increased. There is scope to explore the spatial patterns underlying increased brain-predicted age in different diseases to determine any similarities between diseases. Multiple approaches to evaluating feature (e.g., voxel or brain region) importance in machine-learning models of neuroimaging data exist, including weight-vector mapping, sensitivity mapping and analytically approximated permutations [64, 65]. Recently, Varikuti et al. [66] used an orthonormal non-negative matrix factorisation approach combined with LASSO regression to determine which grey matter regions most influenced age prediction. They found that the majority of cortical and subcortical areas were involved in age prediction, suggesting much of the brain is affected by the ageing process. Interestingly, the exact spatial patterns of feature importance varied across prediction models, which had used different training datasets. This suggests that there are potentially multiple solutions to the problem of predicting age from volumetric MRI data, making it difficult to isolate a universal brain ageing ‘signature’. This motivates research into methods for identifying an individual’s spatial patterns of brain ageing (Box 2). This should enable examination of what factors relate to specific brain-ageing patterns, allowing better evaluation of whether different diseases can result in similar brain changes, along the ageing spectrum. If brain ageing is a global phenomenon [67], the brain’s interconnectedness could mean that similar global changes occur downstream as a result of spatially disparate initial insults, even in diseases with focal brain damage. This interconnectedness is not only at the level of brain structure and function, but also at molecular and cellular scales, with putative ‘molecular nexopathies’ [68], Wallerian degeneration and circulating immune factors (e.g., cytokines) providing potential mechanisms whereby local damage could precipitate global changes that, arguably, resemble those seen in ageing.

Importantly, measures of brain ageing in mild, prodromal disease stages can predict the progression to further cognitive decline and dementia. This implies that a more comprehensive picture of the disease process could be gained by combining more specific measures that reflect distinct pathologies with more general measures of the brain ageing process.

## Bodily ageing biomarkers

The introduction of neuroscientific data into the search for ageing biomarkers is relatively recent. The majority of other research has focused on biochemical measures, often derived from blood samples, as recently reviewed by Jylhävä et al. [69]. Alongside biochemical measures, physiological measures of biological ageing have also shown considerable promise, representing the outward phenotypic manifestation of underlying age-related biological changes. Many potential ageing biomarkers have been proposed, and here we provide an overview of leading candidates. Our intention here is to sketch broadly the conceptual landscape in which brain ageing resides.

## Telomere length and ageing

Telomeres are the nucleo-protein complexes present at the end of all eukaryotic chromosomes. As they shorten each time a somatic cell divides [70, 71], telomere length is often considered an ageing biomarker [72]. Telomere shortening, commonly measured in leukocytes, is associated with a number of environmental factors, including socio-economic status, smoking, oxidative stress, and psychological stress [73–75]. However, the correlation between telomere length and chronological age was shown to be about *r* = −0.3 in a systematic review [76], a weaker relationship with age than other candidates, such as the epigenetic clock, as highlighted by Jylhävä et al. [69].

Over recent years, an increasing body of evidence has suggested that telomere length predicts a small amount of variation in brain, cognitive, and other physiological functions and ageing. Shorter leukocyte telomere length has been linked to: subcortical atrophy (beta = −0.217) and white matter hyperintensities (WMHs) (telomeres of individuals with subcortical WMHs were 371 bp shorter) in a sample of 102 non-demented older individuals [77]; global and regional brain volume in a cohort of 1960 middle-aged individuals (betas = 0.04–0.08) [78]; hippocampal volume in 47 middle-aged women (*R*^2^ = 0.28–0.50) [79]. However, a meta-analysis of telomere length and hippocampal volume, including 2107 individuals, failed to find a significant association [80]. Such findings mean that the relationship between molecular senescence and brain structure remains unclear.

Individual cohort studies of the association between telomere length and cognitive ability have also proved equivocal. A meta-analysis of 12 European cohorts (*N* = 17,052, mean age = 59.2 ±8.8 years), which included Mendelian Randomisation to investigate causation, concluded that longer telomeres result in better cognitive performance, although not all findings withstood multiple testing correction or replication [81]. There are fewer studies that have investigated telomere length and longitudinal cognitive decline. However, our work with the Lothian Birth Cohorts from 1921 and 1936 allowed us to test this association [30]. These general population cohorts included a total *N* of ~1500 older individuals at baseline. We found that although both telomere length and cognitive ability decreased with age, they did so independently, and telomere length at baseline was not associated with cognitive decline [82]. However, a meta-analysis of 13 studies (860 patients and 2022 controls) found consistent evidence of shorter telomeres in Alzheimer’s disease patients (standardised mean difference = −0.984) [83].

Telomere length has also been suggested as a biomarker of other neuropsychiatric disorders including MDD, schizophrenia and bipolar disorder. A meta-analysis of 14,827 individuals indicated that shorter telomere length is associated with a diagnosis of a psychiatric condition (Hedge’s *g* = −0.50) [84].

Shorter telomere length has repeatedly been associated with increased mortality, and in a large cohort study (*N* = 64,637) was associated with increased all-cause mortality (hazard ratio = 1.40), cancer mortality (hazard ratio = 1.52), and cardiovascular mortality (hazard ratio = 1.52) [85]. However, there is little evidence for telomere length as a biomarker of physical decline. Meta-analyses of a number of physical traits (walking and chair-rise speed, standing balance time, grip strength) with sample sizes ranging from 1217 to 3707 found only very weak associations between any of the measures and telomere length [86].

Currently, evidence clearly shows that telomere length declines with age and has some association with mortality and neuropsychiatric disorders. However, it is not a highly predictive biomarker of ageing, thus its use as a measure of biological age at an individual level is not well supported by available data.

## DNA methylation and ageing

While the underlying genetic sequence remains stable over the life course, epigenetic marks, such as DNA methylation—the addition of a methyl group to a cytosine nucleotide in a cytosine–phosphate–guanine pair (CpG)—are dynamic and influenced by both genes and the environment. Microarray technology now enables analysis of methylation at upwards of 500,000 CpG sites across the genome. DNA methylation appears to vary with age, and thus methylation data have been used an ageing biomarker. DNA methylation patterns can be used to predict accurately an individual’s chronological age, as first shown by Hannum et al. [87], and by Horvath [11]; so-called ‘epigenetic clocks’. By using penalised regression methods, Hannum and colleagues derived a 71 CpG-site methylation signature based on 482 whole blood samples that correlated 0.91 with chronological age in 174 independent samples. Horvath derived a 353 CpG-site signature based on over 8000 samples from 51 tissue types to provide a multi-tissue predictor that correlated 0.96 with chronological age in independent samples. DNA methylation can be studied using DNA derived from any type of nuclear cell, and whereas the majority of extant research has used blood samples, DNA methylation age is not necessarily consistent across tissue types within an individual [11].

Recently, Horvath proposed updated versions of both the ‘Hannum’ and ‘Horvath’ clocks that incorporate additional information from age-associated white blood cell counts, which can be imputed from methylation array data [88]. Other, more parsimonious clocks have also been built, for example, a 3 CpG-site clock was derived from a pyrosequencing approach in place of array data [89]. There are also strong correlations (*r* > 0.9) with individual CpG sites and chronological age, such as cg16867657 in the *ELOVL2* gene [90].

Typically, epigenetic epidemiology studies consider both the Hannum and Horvath versions of the epigenetic clock. The biomarker metric used in these studies has been dubbed ‘epigenetic age acceleration’ and is either the raw difference between an individual’s DNA-methylation-predicted age (akin to brain-PAD), or the residual from the regression of DNA-methylation-predicted age on chronological age.

Statistically significant associations between epigenetic age have been reported in a variety of contexts. These include our work on longevity, whereby having a 5-year increase in epigenetic age was associated with 21% increased mortality risk [91]. We also previously found that older epigenetic age was associated with poorer cognitive ability (standardised beta = −0.07), grip strength (standardised beta = −0.05), lung function (standardised beta = −0.06) and walking speed (standardised beta = 0.03) [92]. Other researchers have reported epigenetic age associations with obesity [93], HIV [94], Down’s syndrome [95], and Alzheimer’s disease pathology [96]. In all instances, a ‘faster running’ epigenetic clock—methylation age older than chronological age—was associated with poorer health and function. Men typically have higher epigenetic ages than women by around 1 year [11, 87]. The heritability of both Hannum and Horvath age acceleration is around 40% [91].

Finally, epigenetic outlier burden—the number of times an individual’s methylation levels are more than three times the inter-quartile range below or above the 25th or 75th percentile of a CpG in the population—has been shown to correlate with chronological age, whereby more outliers accumulate over time [97]. How this burden relates to epigenetic clock measures or a broad spectrum of age-associated measures is yet to be investigated.

## Lipids biomarkers of ageing

Lipids are highly prevalent in the brain and are important constituents of cell membranes and also act as signalling molecules [98]. Recent advances in mass spectrometry mean that hundreds to thousands of lipids can now be measured in blood plasma or serum [98, 99]. Studies indicate that changes in lipid metabolism are detectable in Alzheimer’s patients and may predict subsequent cognitive decline, making them a promising biomarker for AD [100, 101]. Sphingolipids, which are particularly abundant in the brain, have been associated with variation in memory impairment (OR = 0.31) and brain white matter structure (betas = 0.17–15.7) in older non-demented individuals, suggesting that they may also be a biomarker of normal cognitive ageing [102, 103]. Lipidomics is in its infancy, but is a promising area of research which may lead to the development of reliable biomarkers for AD and normal cognitive ageing.

## Protein glycosylation biomarkers of ageing

Protein structure, which is defined by DNA sequences, does not change with age. However, glycans are also important constituents of most proteins. They are the product of complex pathways that involve many different proteins and are encoded in complex dynamic networks of hundreds of genes. Glycosylation of proteins does change with age, explaining up to 58% of the variance in chronological age [104]. GlycanAge also correlates with a number of biochemical and physiological age-related measures (betas = 0.0002–2.97) [104].

## Physiological measures of ageing

Alongside biomarkers measuring internal ageing-related processes, a number of external measures of physiological properties of the human body have been associated with ageing. These include measures of body composition (e.g., body-mass index [BMI], waist–hip ratio, bone mineral density, fat mass, muscle mass), and measures of physiological (e.g., blood pressure, heart rate, grip strength, lung capacity, walking speed) and sensory function (e.g., visual acuity, hearing, smell). Generally, these measures consistently show significant, but relatively moderate, correlations with chronological age in healthy people [105–110]. The common approach to measuring these physiological properties generates a univariate measure. The use of univariate measures to predict chronological age is limited by the wide variability often seen at any one age, even in healthy individuals. In this sense, they may be less suitable as precise ageing biomarkers compared to higher-dimensional alternatives (e.g., spatial data on brain structure, DNA methylation status across multiple CpG sites), which perhaps better capture patterns of variability across narrow age ranges. Nevertheless, measures of physiological function have been used to form composite predictive models of biological age. For example, a multivariate model of visual and auditory acuity, grip strength, peak expiratory flow, blood pressure, and BMI was shown to correlate with chronological age (*r* = 0.48) [111]. More recently, physiological measures have been combined with biochemical laboratory measures of biological age, as discussed below.

Interestingly, despite only showing moderate relationships with ageing, physiological measures have been shown to strongly relate to future health. Taking the example of grip strength, results consistently support the promise of this as a marker of general health, robustly relating to disability, morbidity and mortality [112–114]. This raises a fundamental question for biogerontology. Why attempt to measure biological age per se, when one can go more directly to important health outcomes? One answer to this question is that building predictors of the underlying ageing process using biomarkers might better help us understand the mechanisms involved, which in turn could lead to treatments and interventions to help improve health during ageing. However, to properly model cause and effects within the complex interplay of different ageing processes, integrating measures from multiple biological systems will be necessary (see Box 2).

## Integrating measures of biological ageing

Given the evident complexity of the ageing process, it is unlikely that any single measure will be the optimal ageing biomarker. Furthermore, although there is some generality in overall biological ageing, there is also system specificity. This means that change in an individual biomarker may well not indicate increased risk of disease or functional decline [115]. Hence, research efforts have focused on identifying panels of healthy ageing biomarkers [116], to derive composite measures that combine an array of complementary measures of biological properties. This statistical approach often includes biochemical markers, including ‘omics data, with indices of body composition and physiological functioning, and is becoming increasingly popular [37, 117–122]. However, many of these approaches still lack specificity and fail to model the heterogeneity in the biological ageing process within an individual. This heterogeneity has been conceptualised as the ‘mosaic’ of ageing [123], whereby different systems, tissues or cells in a single individual could be undergoing ageing-related changes at different rates. In some senses, this is the antithesis of the common-cause hypothesis [124], which suggests that there is a single underlying factor driving the phenotypic manifestations of ageing. The truth may well lie somewhere between these two theories. While there is often some correlation between different age-related phenotypes [111, 124], this is not universal. In fact, DNA-methylation age predictions from different tissues within the same person are not identical [11]. Furthermore, our combined study of the epigenetic clock and brain-predicted age showed no correlation between blood-derived DNA-methylation age and brain-PAD (rho = 0.001). Whereas the absence of a correlation could be due to measurement error, and is not unequivocal evidence of no relationship, interestingly we observed that both DNA-methylation age and brain-PAD were independently related to mortality risk. Statistically, brain-PAD explained significantly more variance than DNA-methylation age (area under curve [AUC] = 0.66 vs. 0.59), although the most explanatory model combined both measures (AUC = 0.69). This suggests that epigenetic ageing in blood may not have a causal role in structural ageing in the brain, or vice versa, yet when individuals appear older on both counts, mortality risk is heightened.

Our interpretation is that differential ageing rates can occur within an individual. Importantly, alongside this, there are latent systemic mechanisms and bi-directional feedback loops at work [125], meaning that increased accumulation of age-related damage in one system may propagate to another. To better understand this, it will be essential to incorporate data from as many different biological systems as possible. However, judicious statistical approaches will be required to extract meaningful variance and limit the potentially exponential growth in the number of variables. These approaches will include feature selection and dimension reduction methods (e.g., feature importance metrics and principal component analysis, respectively). This will help ensure that dimensionality can be equated across modalities (e.g., when combining brain imaging data, ‘omics data, epigenetic data, and physiological data), so that undue weight is not placed on a single data source. Furthermore, incorporating latent factors and modelling their moderating or mediating influences (e.g., using structural equation modelling) will allow hypotheses regarding cause-and-effect in biological ageing to be tested, helping us understand whether abnormal ageing in one organ or tissue can drive ageing in others.

Some open question about ageing biomarkers remain. Firstly, what does accelerated ageing mean at different points over the life course? Our review has focused on adulthood, with the assumption that appearing biologically older is negative. The consequences of accelerated ageing during childhood-to-adulthood development, however, are unclear and warrant further study. Secondly, why use ageing biomarkers when one could directly relate more straightforward brain structural measure(s) to behaviour or health outcomes? In fact, our study of brain structure and mortality showed that measures of grey matter and cerebrospinal fluid volume were more strongly related to mortality than the brain-ageing biomarker, brain-PAD [32]. However, ageing biomarkers have potential utility that other measures do not. In addition to the rationale outline in Box 1, ageing biomarkers allow individuals to be placed in the context of a wider population, giving an idea of whether or not their brain, for example, is similar to what is typical for their age. It also allows some inference about trajectories to be made from cross-sectional data, whereby a higher brain-PAD after a brain injury, for example, suggests that a negative progressive process has been triggered by that injury [52]. Biological age also has the potential to be measured in younger adults, when the confounds of chronic disease are lower and the likelihood of successful strategies to reduce age-related morbidity is greater [119]. Finally, the unit of measurement of ageing biomarkers is intuitive and can summarise complex information in a comprehendible manner. For example, a patient may more easily understand the connotations of having an older-appearing brain or a faster-running epigenetic clock than they would being told that their brain is a specific number of mL in volume or that their CpG sites at specific genomic locations are differentially methylated. Only further studies can settle these open questions; however, research into the biology of ageing should be conducted mindful of these issues.

## Conclusions

Over recent years, neuroimaging data have been increasingly used to model healthy brain ageing. These efforts have been conducted in parallel to, but rarely in combination with, research into ageing biomarkers derived from measures of blood chemistry, body composition, or physiological functioning. This neuroimaging research has shown that psychiatric and neurological diseases can influence the brain-ageing process, as can non-CNS conditions. Alongside this, neuroimaging measures of brain-predicted age can provide prognostic information about the progression of individuals to cognitive decline, dementia, and subsequent death. In our view, this suggests that some of the long-term downstream sequelae of different brain diseases may overlap with each other and with the changes to brain structure seen during ageing. Potentially, a shift towards a greater emphasis in research on measuring individual differences, rather than group-average characteristics, will provide better predictions for long-term health outcomes in brain diseases and more generally.

However, whereas neuroimaging studies of brain ageing are informative and potentially useful in a clinical setting, basic mechanistic studies should follow, to uncover the molecular and cellular processes driving these phenotypic alterations. This should help us better understand whether fundamentally age-related processes are occurring, or whether the commonalities between disease and ageing are in fact epiphenomena. Epiphenomena or not, the brain-predicted age measure appears to meet all the same criteria for an ageing biomarker as other measures, such as the epigenetic clock. Telomere length, despite its long-standing popularity, appears, in fact, to be less appropriate than brain-predicted age, either at predicting chronological age or health outcomes. The long-term goal of biogerontology should be to integrate the measurements of as many age-related epiphenomena as possible, using the growing array of biological measurement techniques available. It is notable that, in a number of recent reviews of ageing biomarkers, neuroimaging studies and in fact the brain in general, are overlooked [69, 126, 127]. To develop robust, reliable and valid ageing biomarkers that are truly integrative across the human biological system, it is time to blend biogerontology with neuroscience in efforts to understand and improve health during ageing.
